# The Analysis of Job Satisfaction of Health Practitioners in Saudi Arabia: Determinants and Strategic Recommendations for Health Workforce Planning

**DOI:** 10.7759/cureus.50891

**Published:** 2023-12-21

**Authors:** Talal Alotaibi, Mohammed Alsahafi, Osama Alariany, Ayman A Alqarni, Maha Abuzenada, Eman Almutairi, Ziad Nakshabandi, Fahad Alyami, Basim Alsaywid, Miltiades Lytras

**Affiliations:** 1 College of Medicine, King Abdulaziz University Faculty of Medicine, Jeddah, SAU; 2 Research and Development, Saudi Commission for Health Specialties, Jeddah, SAU; 3 Surgery, King Saud University, Riyadh, SAU; 4 Computer Science, Effat College of Engineering, Effat University, Jeddah, SAU

**Keywords:** policy making, digital transformation, saudi arabia, workforce planning, health practitioners, job satisfaction

## Abstract

Background: Job satisfaction plays an important foundation in the health system to deliver high-quality care services to patients. Job satisfaction describes the inside feeling of workers about their jobs whether they like it or not. Job satisfaction of health practitioners is considered an essential factor that affects their efficiency, well-being, and mental health.

Aim: This research study is part of an integrated research aiming to understand the determinants of a revised Workforce Planning Strategy in Saudi Arabia, in close relevance to the demand and supply side in Saudi Arabia.

Results: This study showed that males were significantly more satisfied than females in relation to specialty, organization, and overall global score. There is also evidence for a significant association between age groups and job satisfaction. Older health practitioners had a higher level of satisfaction than younger ones. Regarding nationality, we found that Saudi health practitioners were less satisfied in all aspects, while non-Saudi (Arab) healthcare practitioners were the most satisfied in all aspects except city happiness.

Conclusion: Our study found statistically significant differences that medicine and surgery practitioners are the most satisfied professionals and the least satisfied are dentist practitioners. Health practitioners with more than five years of experience were significantly the most satisfied.

## Introduction

Job satisfaction has a critical effect on the sustainability of the healthcare workforce and workforce planning. Exhausted health practitioners physically and emotionally will provide lower quality of care to the patients [1]. Furthermore, it can be affected by several factors that include, but are not limited to, work hours, workload, relationship with colleagues and supervisors, income, and the patient's respect for the health practitioners [2]. A previous study measured the job satisfaction among general practitioners (GPs) in seven different countries, and it showed that German GPs were least satisfied with work hours and work tasks compared to other GPs of other countries [3]. Another study done in Canada showed that the rate of satisfaction with “professional lives” was 72%, and “work-life balance” reported 49% satisfaction; also, older physicians had twice more satisfaction than younger physicians [4]. In addition, a survey-based study from China found that the average score of job satisfaction was 19.61 out of 30 points, which is considered moderately low [5]. Moreover, another survey-based study from an academic institution in India looked at the job satisfaction of physicians, nurses, technicians, and supporting staff, and concluded that nurses had the highest job satisfaction followed by physicians then supporting staff then technicians [5].

A study from Egypt found that physicians were dissatisfied with income, fringe benefits, and contingent rewards; on the other hand, marriage, and long years of experience correlate with high satisfaction [6]. Another study from Kuwait showed that from a total of 89 participants, the overall job satisfaction was 61.8 out of 100. They also found a high satisfaction rate with increasing age and physicians were dissatisfied with salary and work tasks [7]. In a recent study done in the western region of Saudi Arabia, lifestyle had 63% satisfaction and 25.2% satisfaction with salary. On the other hand, 83.2% were dissatisfied with “contingent rewards” [8]. Another study compares job satisfaction between different medical specialties. The results showed that 66.1% of the physicians were satisfied with the nature of the job, compared to 33.9% who were dissatisfied. Internal medicine and surgical specialties had better satisfaction compared to other medical specialties [9].

The analysis of psychological work factors and job satisfaction appears to be a meaningful and sustainable research path in healthcare. In our research, we focus on the psychological determinants of job satisfaction, and we are willing to interpret the key findings with justified recommendations for Health Workforce Planning in Saudi Arabia. From a workforce planning point of view, all the mentioned factors and variables help us to put together a national action plan for the improvement and enhancement of healthcare workers' well-being and incorporate it into the workforce planning strategy. Our research study focuses on factors that have an impact on job satisfaction and its impact on healthcare workers in Saudi Arabia.

## Materials and methods

This is an observational analytical cross-sectional study, and the survey was distributed to all health practitioners working in Saudi Arabia in 2021. The targeted populations were physicians, nurses, pharmacists, dentists, and applied health professionals without gender disparity and across all regions in Saudi Arabia. Every healthcare professional was included in the study, those who did not respond and refused to participate were excluded.

The data were collected using a self-designed questionnaire with close-ended questions validated by the Saudi Commission for Health Specialities (SCFHS) and distributed as a Google form. The questionnaire has three parts. The first part included demographic questions such as age, gender, marital status, nationality, region, specialty, and income satisfaction. The second part of the survey was a global score towards specialty, organization, city, income, and overall job satisfaction. The score was from (0-10), as “0” is the least satisfied and “10” is the most. The third part included a 20-item survey to measure job satisfaction levels, including relationships with management and supervisors, job content, and relationships with coworkers. The consent was taken as a statement in the questionnaire as a (agree/disagree) question.

The survey was distributed through e-mails to all registered healthcare workers in Saudi Arabia. A total of fifty-thousand e-mails were sent to the targeted population, and 2002 respondents were collected and analyzed. All data are held in strict confidentiality. In our study, descriptive analysis is reported as frequency and percentage for qualitative data. Also, for bivariate analysis chi-square test is used with a level of significant 0.05, and for multivariate data we used analysis of variance (ANOVA) with a level of significant 0.05.

This study was approved by the Institutional Review Board Committee of the Saudi Commission for Health Specialties under the number "SRP-000146".

## Results

**Figure 1:**
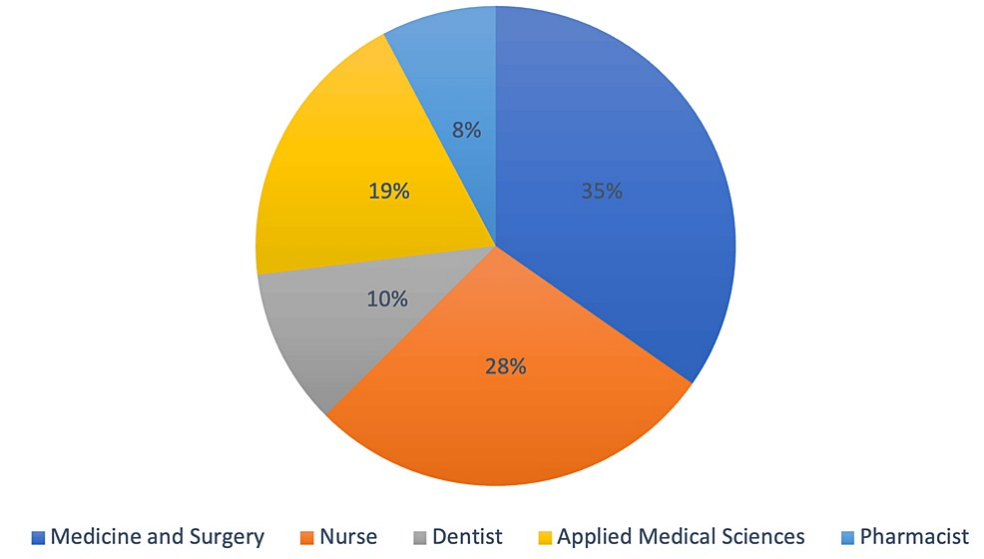
Demographic details of the profession

The participants of our study sample (n = 2002) were healthcare practitioners in Saudi Arabia. The males were more than females (53.5%). For the marital status, more than half were married (69.6%). For nationality, non-Saudi (other) were the highest participants (37.8%), and non-Saudi (Arab) was the least (26.9%). The participants working in the Ministry of Health were the most representative category (44.6%) followed by the private sector (34.6%) and security forces were the lowest (2.2%). When it comes to profession as shown in Figure 1, more than one-third were Medicine and Surgery (34.7%) and the Pharmacists were the least (7.7%).

The majority of the participants' age group was 30-39 years old (39.4%) whereas the least age group was 60 or older (5.6%). Most of the participants were from Riyadh (33.3%) followed by Makkah (19.6%) and Northern Borders being the least (1.2%). Regarding years of experience, more than five years were the highest (74.2%) and less than one year was the lowest (5.3%). When it comes to the job title, the specialist was more than a third of the participants (40.5%). The participants’ income in the range of 5000-9999 SAR being the highest (31.4%) and >30000 SR the lowest (9.8%).

As per global scores (Table 1), city happiness was the highest (median=10) while income happiness was the lowest (median=7). Job satisfaction survey questions are expressed in Table 2.

**Table 1:** Global scores

Global scores	Numbers (N=2002)	Median (1-11)
Are you happy with your specialty	2002	9
Are you happy with your organization	2002	8
Are you happy with your city	2002	10
Are you happy with your income	2002	7
Overall job satisfaction	2002	8

**Table 2:** Job satisfaction survey questions

	N	Mean	Std. Deviation
1. The management of this organization is supportive of me.	2002	66.08	21.58
2. I receive the right amount of support and guidance from my direct supervisor.	2002	66.83	21.81
3. I am provided with all trainings necessary for me to perform my job.	2002	66.91	21.34
4. I have learned many new job skills in this position.	2002	71.29	19.81
5. I feel encouraged by my supervisor to offer suggestions and improvements.	2002	66.12	21.72
6. The management makes changes based on my suggestions and feedback.	2002	60.55	21.59
7. I am appropriately recognized when I perform well at my regular work duties.	2002	65.36	21.31
8. The organization rules make it easy for me to do a good job.	2002	63.81	21.48
9. I am satisfied with my chances for promotion.	2002	60.28	22.09
10. I have adequate opportunities to develop my professional skills.	2002	64.47	21.28
11. I have an accurate written job description.	2002	68.37	20.35
12. The amount of work I am expected to finish each week is reasonable.	2002	67.38	20.54
13. My work assignments are always clearly explained to me.	2002	67.64	19.53
14. My work is evaluated based on a fair system of performance standards.	2002	64.41	21.40
15. My department provides all the equipment, supplies, and resources necessary for me to perform my duties.	2002	65.08	21.49
16. The buildings, grounds, and layout of this facility are adequate for me to perform my duties.	2002	68.43	20.74
17. My coworkers and I work well together.	2002	75.59	18.09
18. I feel I can easily communicate with members from all levels of this organization.	2002	67.84	21.35
19. I would recommend this health facility to other workers as a good place to work.	2002	67.59	24.15
20. How would you rate this health facility as a place to work on a scale of 1 (the worst) to 10 (the best)?	2002	61.75	26.90
Overall score	2002	66.47	15.85

**Figure 2:**
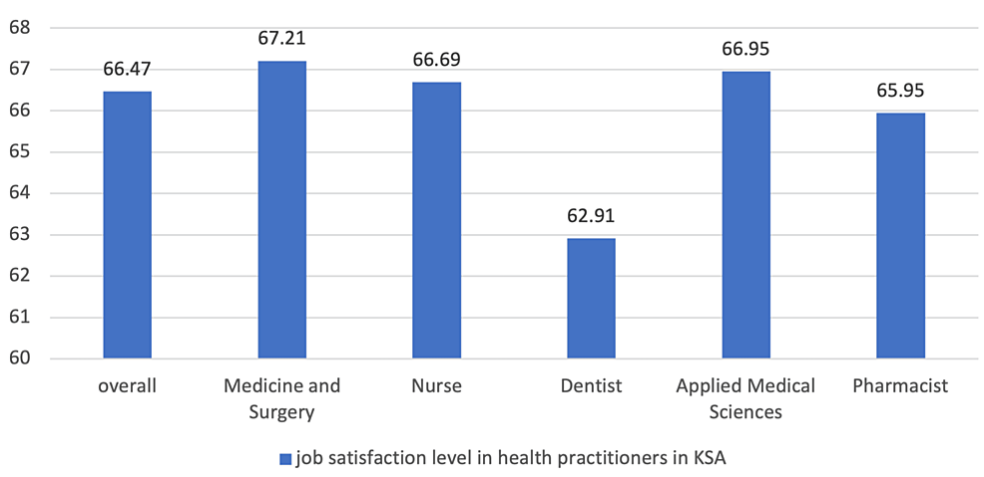
Job satisfaction level in health practitioners in the Kingdom of Saudi Arabia Y-axis = number of satisfied respondents (percentage)

There is a significant difference in overall score and global scores for health practitioners in Saudi Arabia and it shows an overall job satisfaction mean of 66.5 and a standard deviation of 15.85. Medicine and Surgery practitioners are the most satisfied profession with a mean of 67.21, and Dentist practitioners the least satisfied profession with a mean of 62.91 (Figure 2).

**Figure 3:**
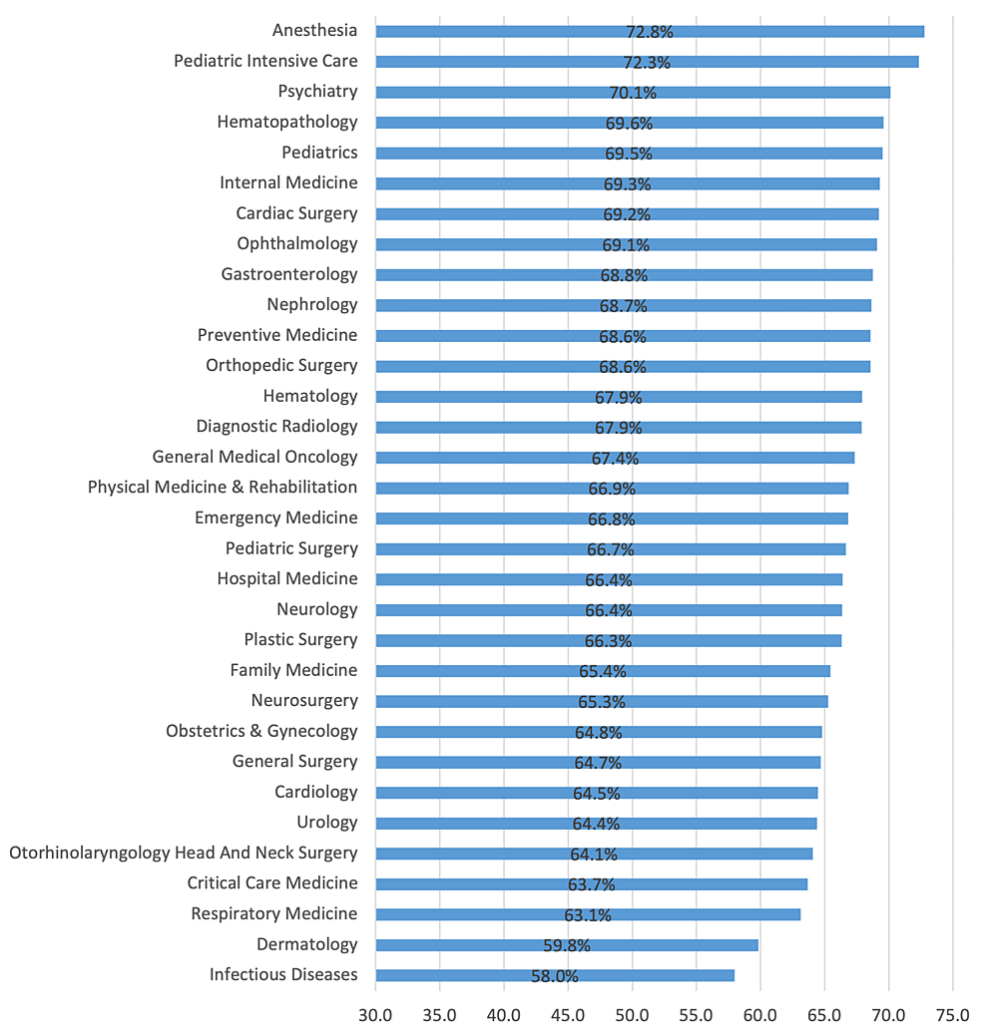
Medicine and surgery specialties satisfaction in Saudi Arabia

As shown in Figure 3, Anesthesia followed by Pediatric Intensive Care were the most satisfied specialties in Medicine and Surgery. The least satisfied specialties were Infectious Diseases and Dermatology.

**Figure 4:**
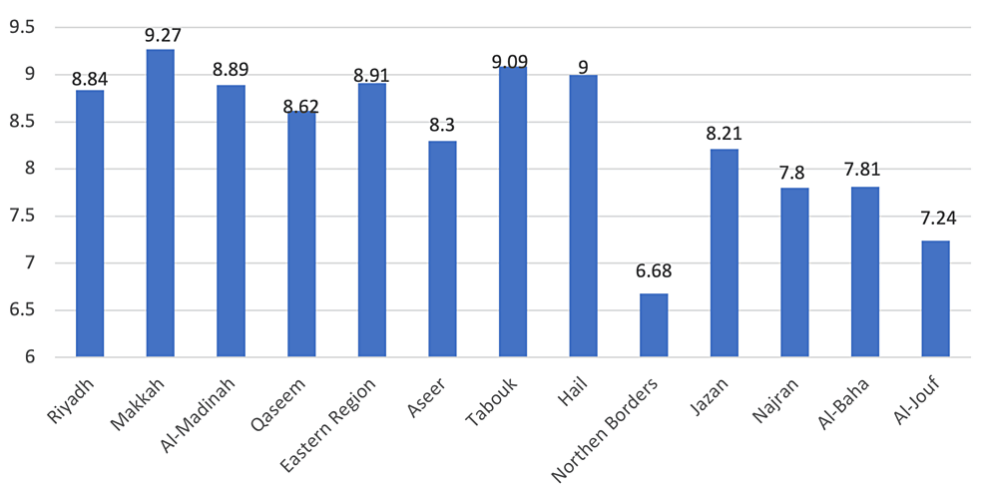
Health practitioners’ city satisfaction Y-axis= mean number of satisfied respondents

As per region, there were no significant differences in overall score and global scores except in city happiness there was a significant difference. Moreover, it shows healthcare practitioners in the Makkah region were the most satisfied with their city of practice and Northern border were the least satisfied with their city of practice (Figure 4).

The overall satisfaction for the gender was not significant (P value= .179). Males were more satisfied in specialty, organization, and overall global scores (P value= .032), (P value= .025), (P value= .001) respectively, while on city and income global scores there were no significant differences between males and females. There is also a significant difference between different age groups in overall scores as well as global scores. Participants aged 60 or older were more satisfied with their jobs in all aspects and in contrast to younger age groups, the satisfaction decreased with the age group 21-29 the least satisfied in all aspects. In terms of marital status, there is a significant difference in overall scores and global scores. Married healthcare practitioners were most satisfied with the overall score (mean=67.6). While the least satisfied are single healthcare practitioners. Regarding nationality, there was a significant difference in overall scores and global scores. Non-Saudi (Arab) healthcare practitioners were the most satisfied in all aspects except in the city happiness aspect non-Saudi (other) were the most satisfied, while Saudi healthcare practitioners were the least satisfied in all aspects.

Regarding the type of facility, healthcare practitioners in Armed Forces facilities were the most satisfied in specialty, income, and overall job satisfaction. While healthcare practitioners in academic institutions were the most satisfied with their city of practice.

We found that practitioners with more than five years of experience are the most satisfied, and practitioners with one to three years of experience are the least satisfied. The global scores are all significant with the same pattern as the overall score, except in city and income happiness where practitioners with less than one year of experience are the least satisfied. Job title of healthcare practitioners shows practitioners with the job title consultant are the most satisfied in specialty, income, and overall job satisfaction.

## Discussion

One of our significant recommendations is that the National Center for Health Workforce Planning and healthcare organizations and institutions should maintain key performance indicators (KPIs) related to healthcare workers' job satisfaction and monitor them over time. Having a biannual or annual overall job satisfaction rate reported will help policymakers and institutions to implement initiatives to improve job satisfaction. It can be linked to the healthcare workers' turnover rate, and this will help having a stable and sustainable healthcare workforce.

Systematic analysis of factors that affect the job satisfaction of practitioners in different health specialties will help to design policies that will improve job satisfaction rates. Given the fact that job satisfaction is also related to job performance, we recommend further investigation of factors that enhance job perceptions and the job satisfaction of health practitioners.

As per region, there were no significant differences in overall score and global scores except in city happiness there was a significant difference. Moreover, it shows healthcare practitioners in the Makkah region were the most satisfied with their cities and the Northern border were the least satisfied with their cities. Thus, it shows the need to understand the factors that help to ensure equal distribution of healthcare practitioners to population ratio in urban vs. rural areas in the Kingdom and to track the turnover rate of practitioners in each region across the Kingdom.

According to our key findings, we concluded that there is lower job satisfaction in younger health practitioners. This can be explained by different factors like job and work-life expectations, resilience, and maturity index. The National Center for Health Workforce Planning believes there is a need to have initiatives to help improving the job satisfaction of younger practitioners. This could include items related to their professional development initiatives, stress management, and work-life balance to enhance their satisfaction and productivity and help them add value. Moreover, in our study, it was evident that nationality is a significant factor in job satisfaction. From this finding, the National Center for Health Workforce Planning believes there is a clear need to investigate further the lower satisfaction rate of Saudi health practitioners, conduct a root cause analysis, and propose solutions with the other stakeholders accordingly.

The research context is complicated since it is related to the job satisfaction of health practitioners, and it is a subjective matter and can be linked to numerous variables. Thus, our study has by default various limitations that challenge the generalization of the findings and the justification of implications. One of the significant limitations is the focus on specific variables that appeared in the theory to be determinants of job satisfaction. Another limitation of our study is the lack of well-established KPIs and metrics for the measurement of job satisfaction for all health specialties. Thus, our approach to quantifying the job satisfaction rate has a risk. In our future research, we will focus on the introduction of new KPIs for all the determinants of job satisfaction that are partially investigated in this research.

The contribution to the body of the theoretical knowledge of the domain is also important. In Table 3, we compare the key findings of our study with key theoretical foundations related to our research.

**Table 3:** Theoretical implications of our study

Key Findings	Comparison with Theory (Other Studies)
Gender: Results showed that males were significantly more satisfied than females in relation to specialty, organization, and overall global score. While there was no significant difference in relation to the overall score, city, and income global score.	Yang Miao et al. showed a significant gender difference with females having higher overall job satisfaction than males [10]. J Paul Leigh et al. and Mohammed Aldossary et al. found no significant difference between male and female satisfaction [11,12].
Age Groups: This study showed a significant association between age groups and job satisfaction. Older health practitioners had a higher level of satisfaction than younger ones.	In contrast to our study, Joanna Kobza did a study among dentists found job satisfaction decreased with increased age [13]. Rebecca Gates and Mubashir Baig Mirza found no significant difference between age and job satisfaction [14,15].
Marital Status: Marital status had a significant difference that showed married health practitioners were the most satisfied group in the overall score and widowed the most satisfied in all global scores. While single health practitioners were the least satisfied.	Other studies done by Abdul Lateef A. Allebdi, Doaa M. Abdel-Salam, Abdulmalik Altaf showed no significant difference between marital status and job satisfaction [8,16,17].
Nationality: Regarding nationality our research found Saudi health practitioners the least satisfied in all aspects, while non-Saudi (Arab) healthcare practitioners were the most satisfied in all aspects except in city happiness non-Saudi (other) were the most satisfied.	Mubashir Baig Mirza found no significant difference between nationality and job satisfaction [15]. Hind I. Al-Haroon conducted a study on nurses and found a significant difference in job satisfaction between Saudi and non-Saudi nurses [18].
Profession: Our study found statically significant differences that Medicine and Surgery practitioners are the most satisfied professions.	Joanna Kobza study showed that 90% of the respondents are satisfied [13]. Hind I. Al-Haroon found that 48% of the nurses were satisfied [18]. Magdalena Iorga found pharmacists were satisfied with the financial budget and were also more satisfied with management–interpersonal relationships and organization-communication, and with their job-total job satisfaction [19]. Mareike Behmann study showed 64% of physicians were overall satisfied [20].
Type of Facility: In our study, healthcare practitioners’ type of facility showed Armed Forces facilities were the most satisfied in specialty, income, and overall job satisfaction. While healthcare practitioners in Academic institutions were the most satisfied with their cities.	Judith Rosta did repeated studies from 2010 to 2016-2017 on job satisfaction and found specialists in private practice the most satisfied followed by GPs and hospital doctors [21].
Working Experience: In our study health practitioners with more than five years of experience were significantly the most satisfied.	Rebecca Gates study showed providers working 11 to 15 years had higher job satisfaction [14]. Doaa M. Abdel-Salam and Nabila S. Ben Slimane showed no significant difference between job satisfaction and years of experience [16,22].
Job Title: This study practitioners with job title consultants are the most satisfied in specialty, income, and overall job satisfaction. Practitioners with the job title assistant consultant are the most satisfied with their organization.	Abdulmalik Altaf found that specialists were less satisfied than consultants [17]. Turki Aldrees showed that 30% of the dissatisfied physicians and 61% of them were consultants [23].
Specialty: In our study anesthesia practitioners were the most satisfied specialty.	Muhammad A. Tobaiqi study found the most satisfied specialty was internal medicine followed by surgery [9]. Doaa M. Abdel-Salam's study showed Clinical Pathology and Radiology were the most satisfied specialties [16].
Income: Regarding income our study found practitioners who are paid more than 30000 Saudi riyals per month are the most satisfied in all global aspects.	Abdul Lateef A. Allebdi's study showed income as the main domain leading to job dissatisfaction [8]. Hind I. Al-Haroon found a significant difference between income and job satisfaction nurses with salaries ranging from 2500 to 4999 SR were more satisfied than those who earned more [18].

## Conclusions

Our research study summarizes a systematic effort to connect job satisfaction determinants with strategic advising for Health Workforce Planning in Saudi Arabia. Our scientific approach provides significant insights that can be used for a strategic plan aiming to promote efficiency and productivity as well as well-being connected to job satisfaction in the health workforce in Saudi Arabia. Our research study provides significant insights into the determinants of the job satisfaction of health practitioners in Saudi Arabia.

The key theoretical contributions of our research allow creative recommendations and interpretations to enhance strategically the Healthcare Workforce Planning in Saudi Arabia. For sure the key recommendations and the implications for managerial practice can be used as benchmarks for other countries as well.

One of the greatest challenges and implications is related to the development of a platform and a managerial system capable of monitoring and reporting the overall job satisfaction rate among health practitioners. For this purpose, we recommend the establishment of unified and trusted metrics in KSA to measure all the different metrics studied in this research over time. This will allow the healthcare administration and leadership to adjust policies and procedures based on evidence provided by the relevant KPIs.

Our research study looked at significant variables connected to job satisfaction including gender considerations/work-life balance, age, job title, working experience, nationality, type of facility, and income. It is extremely important from the decision and policy-making point of view, the need for the special task force to interpret the key findings in all these areas and to integrate them in new policies and action plans. This will allow an evidence-based strategic update on the Workforce Planning action plan.
